# The value of testicular ultrasound in the prediction of the type and size of testicular tumors

**DOI:** 10.1590/S1677-5538.IBJU.2013.0077

**Published:** 2015

**Authors:** Abraham Shtricker, David Silver, Elias Sorin, Letizia Schreiber, Nachum Katlowitz, Alexander Tsivian, Kalman Katlowitz, Shalva Benjamin, Abraham Ami Sidi

**Affiliations:** 1 Department of Urologic Surgery, Edith Wolfson Medical Center, Sackler school of medicine, University of Tel Aviv, Israel;; 2 Maimonidis Medical Center-NY - Department of Urologic Surgery, New York, NY, USA;; 3 Department of Radiology, Edith Wolfson Medical Center, Sackler school of medicine, University of Tel Aviv, Israel;; 4 Department of Pathology, Edith Wolfson Medical Center, Sackler school of medicine, University of Tel Aviv, Israel;; 5 Staten Island University Hospital-NY - Department of Urologic Surgery, New York, NY, USA

**Keywords:** Ultrasonography, Testis, Neoplasms

## Abstract

**Objectives:**

Ultrasound (US) is often used for the work-up of testicular pathology. The findings may implicate on its management. However, there is only scant data on the correlation between US findings and testicular tumor type and size. Herein, we report on a multicenter study, analyzing these correlations.

**Methods:**

The study included patients who underwent orchiectomy between 2000 and 2010. Their charts were reviewed for US echogeneity, lesion size, pathological dimensions, histology, and the presence of calcifications, fibrosis, necrosis and/or intraepithelial neoplasia. The incidence of these parameters in benign versus malignant lesions and seminomatous germ cell tumors (SGCT) versus nonseminomatous germ cell tumors (NSGCT) was statistically compared.

**Results:**

Eighty five patients fulfilled the inclusion criteria, 71 malignant (43 SGCT, 28 NSGCT) and 14 benign. Sonographic lesions were at least 20% smaller than the pathologically determined dimensions in 21 (25%) patients. The ability of US in estimating the size of malignant tumors was 71%, compared to 100% of benign tumors (p=0.03), with no significant difference between SGCT and NSGCT. Necrosis was more frequent in malignant tumors (p=0.03); hypoechogeneity and fibrosis were more frequent in SGCT than in NSGCT (p=0.002 and 0.04 respectively).

**Conclusions:**

Testis US of malignant lesions underestimates the size in 25% of the cases, a fact that may impact on the decision of testicular sparing surgery. The ultrasonic lesions were eventually proven to be benign in 16% of the cases. Therefore it is advised to apply frozen sections in borderline cases. Hypoechogeneity is more frequent in SGCT than NSGCT.

## INTRODUCTION

Ultrasound (US) is often used for clinical investigation of testicular disease. It has a high sensitivity for detection of abnormalities in the scrotum, capable of differentiating between testicular and paratesticular lesions and is accurate in demonstrating the location and characteristics of intratesticular lesions. However, it cannot reliably differentiate benign from malignant intratesticular lesions and its ability to predict the true tumor size is debatable (1-5). It has been demonstrated that cancers are hypo-echoic in relation to the surrounding parenchyma in approximately 95% of cases (6). Some studies have suggested that seminoma germ cell tumors (SGCT) are often more homogeneously hypoechoic while the more cystic nonseminomatous germ cell tumors (NSGCT) are often non homogenously hypoechoic due to areas of calcifcation and/or necrosis (1, 6, 7). Even with this noted difference, the tumor tissue type cannot be reliably differentiated solely by its ultrasonographic appearance and the general consensus is that a sonographic detection of a solid or mixed cystic lesion mass requires surgical exploration (6, 8). In these situations lesion dimensions are a crucial factor if considering testicular sparing surgery (9-12). There is only scant published data on the correlation between sonographic findings and the anatomical size, local stage, type, and histology of testicular tumors (TT) (8). Our major goal was to assess the ultrasound capability to distinguish benign from malignant disease and to estimate the tumor size as compared to pathological measurements. Herein, we report the results of a multicenter study analyzing these correlations.

## MATERIALS AND METHODS

The study included all patients who underwent an orchiectomy from 2000 to 2010 and had their preoperative sonogram and postoperative pathology available.

The patients' charts were reviewed for sonographic parameters such as echogeneity (hyper, hypo or iso), lesion size, and presence of calcifications as well as pathological parameters such as tumor dimensions (after shrinkage due to formalin fixation), histology, and the presence of fibrosis, necrosis and/or testicular intraepithelial neoplasia (TIN). No centralized review was done. As this is a multicenter study, the sonographic and pathological sizes (accounting for formalin shrinkage) were measured by the radiologists and pathologists at their respective medical centers. We defined two sets of tumors: malignant vs. benign tumors and, within malignant tumors, SGCT vs. NSGCT. The Two-tailed Fischer exact test was applied to these sets for all the aforementioned sonographic and pathological parameters.

## RESULTS

There were 85 patients who fulfilled the inclusion criteria, 71 malignant (43 SGCT, 28 NSGCT) and 14 with benign lesions (12 Leydig cell tumor, 1 post traumatic atrophy, and 1 dermoid cyst). Therefore, in 16% of the cases, the ultrasonic lesions were eventually proven to be benign. Lesion dimensions as determined by ultrasound were at least 20% smaller (the minimum difference to be considered in size underestimation in US) than the pathologically determined dimensions in 21 (25%) patients. The results are detailed in Tables 1, 2, 3 and 4.

**Table 1:** Collected ultrasonic results divided according to the type of tumor (benign vs. malignant).

	Benign	Malignant	P Value
Number of pts.	14	71	
US size underestimation	0	21	0.017
Hypoechogeneity	7	32	0.775
Calcifications	4	17	0.739

**Table 2:** Collected histologic parameters divided according to the type of tumor (benign vs. malignant).

	Benign	Malignant	P Value
Number of pts.	14	71	
Fibrosis	2	17	0.726
Necrosis	0	21	0.017
TIN	N/A	34	N/A

**Table 3:** Collected ultrasonic results divided according to the histologic malignant subtype of tumor.

	SGCT	NSGCT	P Value
Number of pts.	43	28	
US size underestimation	11	10	0.429
Hypoechogeneity	28	7	0.001
Calcifications	13	6	0.584

**Table 4:** Collected histologic parameters divided according to the histologic malignant subtype of tumor.

	SGCT	NSGCT	P Value
Number of pts.	43	28	
Fibrosis	14	3	0.047
Necrosis	13	9	1.00
TIN	23	12	1.00

**Figure 1:**
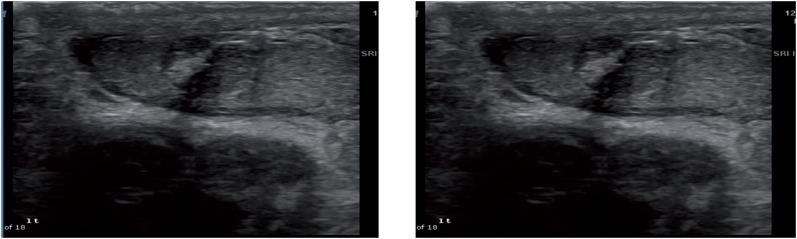
Leydig cell tumor - Heterogeneous mass that was suspected for malignancy eventually found to be Leydig cell tumor with similar size estimated preoperatively.

**Figure 2:**
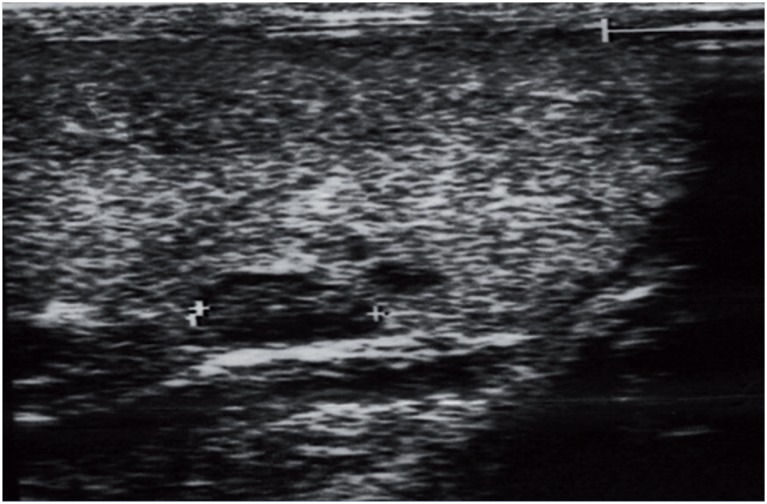
Seminoma - Three hypo and anechoic lesions with variable diameters eventually found to be pure classical seminoma. The rest of the testis was fully indurate by seminoma nests, although homogeneous, non suspected preoperatively. Obviously, this case demonstrates sonographic underestimation of tumor size.

Tumor dimensions measured by sonography were more accurate in benign tumors (p=0.017). The ability of US in estimating the size of malignant tumors was 71%, compared to 100% of benign tumors, with no significant difference between SGCT and NSGCT. We also confirmed that necrosis was more frequent in malignant than benign tumors (p=0.017) and that hypoechogeneity and fibrosis were more frequent in SGCT than in NSGCT (p=0.001 and 0.047 respectively) (Figures 1 and 2).

## COMMENTS

Testicular ultrasonography is usually performed with a high-frequency linear transducer; the echo texture of the two testicles is compared and areas of heterogeneity are searched for. Upon discovery of a lesion accurate dimensioning is crucial as clinicians must carefully consider the size of the lesion in their decision as to whether or not to perform testis preserving surgery, especially when facing a single testis (anatomical or functional) (13). General consensus is that a sonographic finding of any solid or mixed cystic lesion mass is an indication for surgical exploration (6, 8). However, there are only scant publications on the correlation between sonographic findings and type, local stage, size, and the histology of testicular tumors (TT) (8).

Most papers presented the histological subdivision of tumors without specific correlation as to the echogeneity or size (8, 14). If the size was mentioned in order to justify a partial resection, it was without correlating to the preoperative sonographic findings. Carmignani et al. (8) have outlined the relation between lesion dimensions and the presence of germ cell tumors, showing that lesions of 16-32 mm have a high relative risk for malignancy. Unfortunately this study cannot represent the general population of testicular tumors due to its small size of only 48 subjects and inclusion of only patients with impalpable lesions. Shilo et al. presented a larger group of 131 patients concluding that benign lesions tend to be smaller than malignant lesions (15 mm vs. 41 mm respectively) and therefore a proper sonographic estimation can lead to consideration of partial orchiectomy (15). In contrast, our study explored the ability of sonography to predict the actual pathological size in the post operative specimen and not just the correlation between size and malignancy. The few publications that relate to testicular organ sparing operation focus on the oncological point of view but some sonographic data can be retrieved from them (9-11). Heidenreich et al. presented on 73 patients (42 SGCT, 31 NSGCT). Elert et al. operated on 354 patients, revealing 317 tumors: 100 seminomas, 217 nonseminomas, and 14 Leydig cell tumors (12). This large group of patients is impressive yet their data was not used to examine the preoperative sonographic expression and only concentrated on indications for frozen section, organ sparing surgery, and the oncological outcomes. Weissbach mentioned the limitation of size (≤20mm) as one of the indications for partial orchiectomy without estimating the sensitivity of the preoperative sonogram to give such important detail (15). Some authors attempted to test the sonographic utility by searching for a correlation between the sonogram and the postoperative results but did not consider size estimation (16). Wang used data of 59 tumors (41 seminomas, 9 non seminoma and 6 non germ cell tumors) to discuss the ability of the sonogram to differentiate between malignant and nonmalignant lesions (17, 18). Ye et al. presented 16 patients with impalpable masses (diameter of 5 to 30mm) of which 15 were hypoechoic and one was hyperechoic and calcified with only 5 malignant masses (2 seminomas, 1 nonseminoma and 2 cases of lymphoma). This attempt pointed to our goal but the low power of the study precluded any conclusive deductions. Schwerk et al. (6) has reported a prospective study on 57 lesions, demonstrating a broad spectrum of texture patterns for malignancies of which 92% exhibited hypoechogeneity, but could not differentiate between the histological subtypes. There is no doubt that this publication investigates a part of our discussion. However, the number of patients is inferior to our study and their study does not deal with the ability of the sonogram to evaluate the size of the tumor and therefore does not contribute to the planning of partial orchiectomy. Moreover, our data confirms the predominance of hypo-echogeneity in seminoma and therefore contributes to the preoperative evaluation.

Our study provides the percentage of preoperative sonographic tumor size underestimations, an issue not yet addressed. Moreover, we attempt to support prior assumptions presented in urological literature without sufficiently solid proof regarding the ability of sonographic findings to predict testicular tumor type. More than that, we have demonstrated another preoperative tool or attempt to distinguish benign from malignant tumors aside to other characteristics that have been described by Shilo and his colleagues (13).

This new data provides help in the surgical consideration and planning of an orchiectomy, especially the consideration of a partial resection with or without a guided intraoperative biopsy. Herein we are adding another proof for the opinion that seminomas tend to be more hypoechoic than nonseminoma tumors. These facts combine to show that concentrating on the sonographic characteristics of the testicular lesion can vastly improve clinical judgment. A reason for underestimation might be that sonography only shows the centralized body of the malignancy and cannot reliably pick up tendrils that are of clinical significance. To improve preoperative management and characterization of nonvascularized tissue, contrast-enhanced ultrasound, a new sonographic technique, can be performed as an adjuvant to color Doppler ultrasound. Its role in evaluation of malignant suspected lesions is not well defined and therefore not included in the official urological guidelines. The role for elastography, a medical imaging modality that maps the elastic properties of soft tissue, is limited to small testicular lesions, especially in surveillance. However, the combination of these two techniques along with the clinical conclusions of our study might improve the future management of testicular lesions (19).

Before concluding we should note a few points about our study. We made the tradeoff of including more patients thereby increasing the power of our study and accepting the need for the requirement of a non-centralized pathological and radiological review. Additionally, although our patients were heterogeneous, prior publications that contributed to our knowledge of the prevalence of different types of tumors within groups of patients lead to the conclusion that this is acceptable (20,21).

## CONCLUSIONS

Sonographic measurement of malignant testicular lesions underestimates the size in 25% of the cases. This can have serious consequences, as size has an impact on the decision of testicular sparing surgery. For example, a large tumor that compromises most of the parenchyma will preclude an attempt at organ sparing. Intraoperative frozen sections testing for negative surgical margins could/should be used, similar to what is the gold standard in other realms of partial resections such as renal malignancies. This may decrease the known failure rate of about 25% in patients who underwent partial orchiectomy as presented at prior gatherings (AUA 2010). Further studies looking at the correlation between tumor size and the magnitude of underestimation would be useful. Additionally, although the fact that this was a multicenter study decreases the chance that our findings are due to a local lack of training, further confirmatory studies at other sites would be useful. The ultrasonic lesions were eventually proven to be benign in 16% of the cases, and therefore a liberal use of frozen sections in borderline cases is advised. Hypoechogeneity and fibrosis are significantly more frequent in SGCT than NSGCT and necrosis is not present in benign tumors.

## ABBREVIATIONS

TT =: Testicular tumor
TIN =: Testicular Intraepithelial Neoplasia
SGCT =: Seminoma
NSGCT =: Nonseminoma
US =: Ultrasound
